# An evolutionary inspection game with labour unions on small-world networks

**DOI:** 10.1038/srep08881

**Published:** 2015-03-06

**Authors:** Salahuddin M. Kamal, Yas Al-Hadeethi, Fouad A. Abolaban, Fahd M. Al-Marzouki, Matjaž Perc

**Affiliations:** 1 Department of Physics, Faculty of Sciences, King Abdulaziz University, Jeddah, Saudi Arabia; 2 Engineering College, King Abdulaziz University, Jeddah, Saudi Arabia; 3 Faculty of Natural Sciences and Mathematics, University of Maribor, Koroška cesta 160, SI-2000 Maribor, Slovenia; 4 CAMTP – Center for Applied Mathematics and Theoretical Physics, University of Maribor, Krekova 2, SI-2000 Maribor, Slovenia

## Abstract

We study an evolutionary inspection game where agents can chose between working and shirking. The evolutionary process is staged on a small-world network, through which agents compare their incomes and, based on the outcome, decide which strategy to adopt. Moreover, we introduce union members that have certain privileges, of which the extent depends on the bargaining power of the union. We determine how the union affects the overall performance of the firm that employs the agents, and what are its influences on the employees. We find that, depending on its bargaining power, the union has significant leverage to deteriorate the productivity of a firm, and consequently also to lower the long-run benefits of the employees.

How and if at all unions affect labour market relations remain two central questions in the industrial relation research. The work by Freeman and Medoff entitled “What do Unions do?” is thereby, even today, one of the most influential sources since its publication in 1984^1^,^2^. In general, it is known that the primary objective of unions is to enforce the interests of their members. Unfortunately, sometimes these interests have, however well-intended, negative consequences that in the long term benefit neither the firm nor its employees. In this paper, we therefore aim to systematically analyse the impact of labour unions on the performance of firms as well as on the strategies of workers that warrant this performance. And we do so by employing the theoretical framework of evolutionary games^3^,^4^,^5^,^6^,^7^ and network science^8^,^9^,^10^,^11^,^12^,^13^,^14^,^15^,^16^.

At the core of our study is an inspection game proposed by Dresher^17^. Originally, the game comprises a principal (employer or firm) that employs an agent (employee or worker), whereby the principal assigns a task to the agent for which the latter, if successfully accomplished, will receive a payment. Although straightforward at first sight, the arrangement results in the well-known and rather complex principal-agent problem because the two participants have opposite interests^18^. In particular, while the employer wants his task accomplished, the employee tries to receive his payment with as little effort as possible. The dilemma is tackled by a costly inspection, going at the expense of the employer, intended to reveals the true effort of the employee. If the employee is caught shirking he doesn't get paid.

The union is introduced as a potential escape hatch for shirkers, in particular by warranting them a partial pecuniary compensation if being union members. When deciding whether to join the union or not, employees weigh expected benefits with the established union-membership fee^19^. Thereby, the level of the pecuniary compensation directly determines benefits employees can get if joining the union. On the other hand, the level of the pecuniary compensation that the union is able to warrant their members if they are caught shirking also determines the bargaining power of the union. Presently, we analyse how the union affects the overall performance of the firm, and what are the influences on the employees. In accordance with recent studies^20^, we find that under certain conditions the union has quite a negligible impact on the performance of the firm, but also, that raising the union's bargaining power is, in general, negatively correlated with the performance of the firm as well as with the working habits of their employees.

In what follows, we present the results, where we first introduce the evolutionary inspection game with labour unions and then deliver our main conclusions. Lastly, we briefly discuss the implications of our results and present further details about the applied methodology in the Methods section.

## Results

### Evolutionary inspection game with labour unions

**Figure 1:**
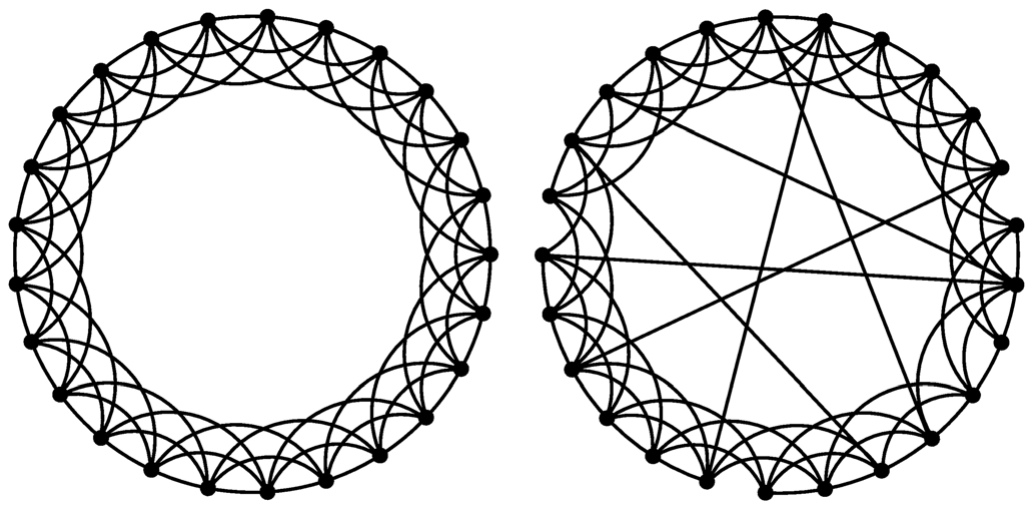
Schematic example of the employed network structure. For clarity regarding the initial degree of each player *k* and the rewiring probability *p* only 25 vertices are displayed in each panel. On the left is a regular ring obtained with *p* = 0 and periodic boundary conditions. Each player is connected to its *k* = 6 nearest neighbours. On the right we have a small-world network, which was obtained by rewiring 6 out of all the 150 edges that form the interaction network. Hence in this case *p* = 0.04.

We first extend the inspection game proposed by Dresher^17^ by introducing structured interactions among employees, and most importantly, allowing them to adjust their behaviour (strategy) in accordance with the performance of their partners. The latter are assigned to each employee via a small-world network generated with the algorithm proposed by Watts and Strogatz^21^, as schematically depicted in Fig. 1. Thus, we introduce an evolutionary process in the inspection game by allowing employees to adjust their working/shirking habits in accordance with the performance of their co-workers. Thereby, the use of the small-world network seems appropriate as it mimics real-life social interactions, which are arguably very influential by labour market issues, for example affecting employment and inequality in notable ways^22^.

The above-introduced evolutionary inspection game thus starts with a principal *P* and a set of agents *A_i_* that are located on vertices of a small-world network. Throughout this work we employ a small-world network consisting of *i* = 1, 2, …, *n* = 1000 vertices with an average connectivity *k* = 6 and rewiring probability *p* = 0.1^21^ (see Fig. 1 for details). Moreover, each agent *A_i_* may choose between two strategies, latter being shirking (*S*) or working (*W*), whereby both strategies are initially uniformly distributed across the network. On the other hand, the principal *P* can opt to inspect (*I*) or not (*N*). Finally, during a single iteration of the game each agent *A_i_* plays the game with the principal *P*, and depending on the employed strategies, the two receive payoffs described succinctly by the payoff matrix:
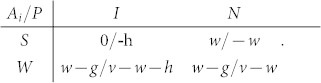


When working, each agent produces the output *v* for the principal, whereby the latter gives the payment *w*. Additionally, each agent that works bears some work-related costs *g*. If *P* decides to inspect it bears and additional cost *h*, but if the agent is found shirking (producing zero output) *P* saves *w*. Importantly, inspection is the only way to find out whether an agent works or not, since it is assumed that *P* cannot condition the wage on the observable outcome *v*. During the game *A_i_* and *P* choose their strategies simultaneously, meaning that they don't know the decision made by the opponent. Additionally, we introduce a probability *r* that *P* will perform an inspection by a given agent *A_i_*. After each full iteration of the game (when *P* interacts with all *A_i_*) agents compare their accumulated payoffs with those of their partners as defined by the small-world network, whereby the probability that an agent *A_i_* adopts the strategy (*S* or *W*) of one of his randomly chosen partners *A_j_* is defined by the Fermi function^23^
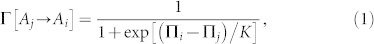
where *K* > 0 is the uncertainty related to the strategy adoption, following the intuition from Selten's “trembling hand perfection”^24^ that the better performing agent is readily adopted while it is also not completely impossible to adopt the strategy of a worse performing player. Moreover, Π*_i_* and Π*_j_* are cumulative payoffs of *A_i_* and *A_j_*, respectively, which for the *t*-th iteration of the game are calculated according to
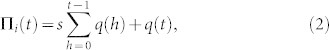
where *s* is the workers savings rate, while *q*(*h*) and *q*(*t*) is the payoff of *A_i_* at iteration time *h* and *t*, respectively.

To study the impact of the union in the evolutionary inspection game, we extended the above basic scheme by introducing a fraction *u* of agents that become members of the union, whereby members of the union are randomly scattered amongst non-members. Consequently, an agent *A_i_* may now have four different strategies, latter being shirking (*S*) and working (*W*), as well as shirking while being a union member (*SU*) and working while being a union member (*WU*). The payoff matrix now takes the form:
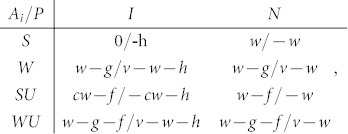
where *c* denotes the level of pecuniary compensation, i.e., the fraction of the whole wage *w* members of the union get even though they were caught by *P* while shirking, and *f* is the union membership fee. All other parameters have the same meaning as by the absence of union members. Importantly, when an agent adopts the strategy of one of his partners, the potential membership in the union is not adopted, meaning that the initial fraction of union members *u* remains constant throughout the simulation.

### Productivity and strategy distributions

**Figure 2:**
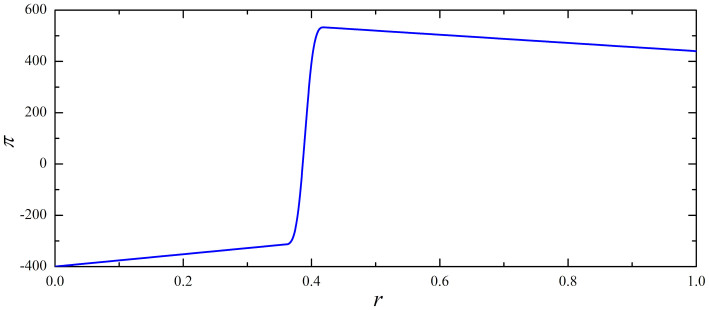
The baseline productivity of the firm in the absence of union members. Depicted is the average income of the firm per iteration Π in dependence on the frequency of inspection *r*. It can be observed that there exists an optimal value of *r* at which the productivity is maximal.

First, we consider the inspection game without union members (*u* = 0). Thus, each agent can either shirk (*S*) or work (*W*), whereby the first payoff matrix introduced above applies. Depending on the probability *r* that the principal *P* will inspect, the average productivity of the firm per iteration Π varies as shown in Fig. 2. It is evident that there exist an optimal value of *r* for which Π is maximal, equalling Π = 536 at *r* = 0.4. If inspections are less frequent some employees may not work, whereas larger values of *r* simply decrease the firm's earnings because of unnecessary and costly inspections. Assuming that by *r* = 1 all employees work, it is straightforward to calculate Π analytically according to Π = *n*(1 − *w* − *h*), which returns Π = 440. Obviously this is less than the optimal outcome obtained by means of simulations on the small-world network (Π = 536) at *r* = 0.4. On the other hand, if *r* = 0 nobody works (and nobody gets caught by the principal *P*), thus Π = *n*(−*w*) = −400. Either way, these limiting cases are in perfect agreement with the simulation results presented in Fig. 2.

**Figure 3:**
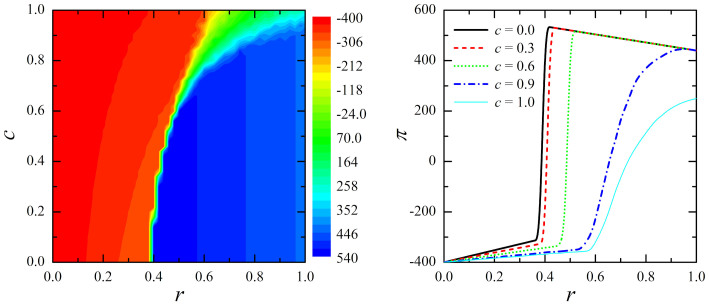
The productivity of the firm in the presence of union members. Left panel shows the colour-encoded average income of the firm per iteration Π in dependence on the frequency of inspection *r* and the level of pecuniary compensation *c*. For clarity, the right panel features Π in dependence on *r* separately for different values of *c* (see legend). Note that for *c* = 0 the curve is the same as shown in Fig. 2. Note that as the value of *c* increases the productivity of the firm decreases. Moreover, the optimal value of *r* increases until it eventually becomes equal to 1. There were a total of 40% union members (*u* = 0.4) among all agents present during the simulation.

Next, we introduce union members to the game by setting *u* = 0–4, whereby the second payoff matrix introduced above applies. Moreover, besides the frequency of inspection *r*, the second crucial parameter is now *c*, which determines the level of pecuniary compensation. Figure 3 shows how the average productivity of the firm per iteration Π varies in dependence on *r* and *c*. It is obvious that for *c* = 0 results converge to the case when union members are absent. Note that *c* = 0 corresponds to the case when the union has no benefits to offer to their members, or alternatively, when it is completely without bargaining power. Then, all limiting cases as well as the peak at *r* = 0.4, equalling Π = 536, are the same as reported in Fig. 2. Thus, it is clear that union presence per se does not influence the labour market, but rather, the influence of the union is directly correlated with its bargaining power *c*. In particular, as the authority of the union increases (*c* → 1), the maximal average income of the firm per iteration (Π) decreases steadily. Thus, by *c* = 1 the maximal values of Π is obtained at *r* = 1, whereby then Π = 250, which is slightly more than 50% lower as the peak value of Π shown in Fig. 2. Obviously, the bargaining power of the union is crucial for its effect on the performance of the firm. As anticipated, the union without bargaining power cannot affect the performance of the firm (but just lowers the net income of their members by the value proportional to the membership firm *f*). On the other hand, as the bargaining power of the union increases, the deterioration of the firm's performance is vast, ultimately resulting in as much as 50% lower net output.

**Figure 4:**
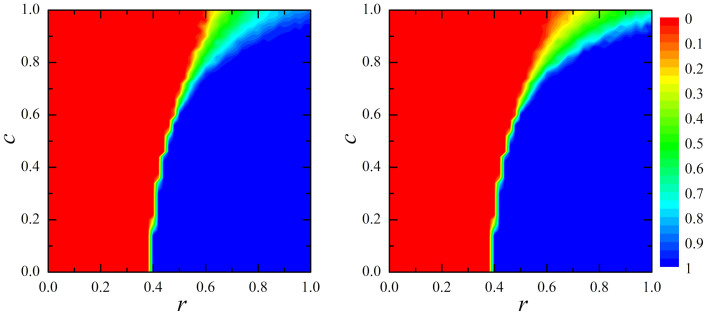
Working habits of the employees based on the membership in the union. Depicted is the colour-encoded fraction of players that work within non-members (left panel) and members (right panel) of the union in dependence on the frequency of inspection *r* and the level of pecuniary compensation *c*. Interestingly, union members decrease the viability of working also among non-members. Other parameter values are the same as in Fig. 3.

Lastly, to analyse the union's influence on the working habits (or adopted strategies *S* or *W*) of agents, we consider members of the union and non-members separately. Figure 4 shows the fraction of agents that work within the two groups (non-members left, members right panel) in dependence on the frequency of inspection *r* and the level of pecuniary compensation *c*. It is fairly obvious that union members are somewhat more prone to shirking than non-members, although the difference is expressed most clearly when *c* → 1. For example, at *c* = 1 and *r* = 1 as much as 90% of non-members work, whilst among union members only 65% choose to work. Remarkably though, and quite surprising as well, an union with substantial bargaining power (a high *c*/*f* ratio) appears to have a deteriorating effect on working habits of both groups of agents (members and non-members), as all are substantially more prone to shirking than in the absence of the union (or equivalently, when the union is completely without bargaining power (*c* = 0)). Thus, the concluding observation that imposes itself is that powerful and influential unions have a negative influence on the working habits of all agents working for the firm (principal), which in turn explains the convincing deterioration of the firm's output and performance, as evidenced in Fig. 3.

## Discussion

We have analysed the influence of unions on the performance of firms and on the strategies employed by their members. We have shown that the union presence per se doesn't have an impact on the output of the firm. However, this statement depends heavily (and logically) on the bargaining power of the union. In particular, we show that if the bargaining power of the union increases, the performance of the firm decreases. Remarkably, a firm with an influential union may face up to 50% lower net incomes than this would be the case in the absence of the union (or equivalently, if the union would have no authority and bargaining power). Thus, it seems reasonable to conclude that unions have considerable leverage to lessen the output of a firm, and consequently also the overall welfare of their employees. Accordingly, we have also found that, albeit in a bit lesser extend, the union has an indirect effect even on non-members, in particular imposing a tendency to shirk when this is neither optimal nor desirable.

It is worth emphasizing that our work is only the first step towards the consideration of unions in the framework of evolutionary games on networks, and in particular in the realm of the evolutionary inspection game. Further research is needed to clarify the subtleties of union effects on the working environment in firms and enterprizes, and we hope that this paper will be motivational to that effect.

## Methods

The studied inspection game with unions is iterated forward in time using a synchronous update scheme, thus letting all agents strike a deal with the principal according to the employed payoff parameters and the probability to inspect *r*, and then simultaneously updating their strategy according to the Fermi function given by Eq. 1, whereby taking into account the performance of one randomly chosen partner constituted by the small-world network. For sufficiently long simulation times the average payoff of the principal per iteration Π, as well as the fractions of strategies *S* and *W* on the network approach an equilibrium value irrespective of the initial conditions. All equilibrium values were calculated after initial transients were discarded, and additionally results were averaged over 20 different realizations of the small-world network and distributions of union members amongst the non-members.

In the simulations, we set the output level of each agent *v* equal to 1, and adjust all other parameter values accordingly. In particular, each working agent earns *w* = 0.4 and bears work-related costs *g* = 0.125. The workers savings rate equals 10% of the wage, thus *s* = 0.04, and the union membership fee equals 5% of the wage, thus *f* = 0.02. Moreover, each inspection costs the principal *h* = 0.16, and the noise level in the Fermi function given by Eq. 1 is *K* = 0.1. Parameters *r* (the probability that *P* will inspect) and *c* (the level of pecuniary compensation) may vary within [0, 1].

## Author Contributions

S.M.K., Y.A.-H. and F.A.A. designed and performed the research. F.M.A.-M. and M.P. designed the research and wrote the paper.
